# Data for the gene expression profiling and alternative splicing events during the chondrogenic differentiation of human cartilage endplate-derived stem cells under hypoxia

**DOI:** 10.1016/j.dib.2016.04.037

**Published:** 2016-04-21

**Authors:** Yuan Yao, Jin Shang, Weilin Song, Qiyue Deng, Huan Liu, Yue. Zhou

**Affiliations:** a Department of Orthopedics, Xinqiao Hospital, Third Military Medical University, Chongqing 400037, China; b Department of Ophthalmology, Southwest Hospital, Third Military Medical University, Chongqing 400038, China; c Department of Neurobiology, College of Basic Medical Sciences, Third Military Medical University, Chongqing 400038, China

## Abstract

This article contains relevant data of the research article titled Global profiling of the gene expression and alternative splicing events during the hypoxia-regulated chondrogenic differentiation in human cartilage endplate-derived stem cells (Yao et al., 2016) [1]. The data show global profiling of the DEGs (Differentially expressed genes) and AS (Alternative splicing) events during the hypoxia-regulated chondrogenesis of CESCs (human cartilage endplate-derived stem cells) by using Affymetrix Human Transcriptome Array 2.0 (HTA 2.0) system. In addition, the enriched GO (Gene Ontology) functions and signaling pathways are listed. The information presented here includes the information of patients from which the clinical samples are obtained, the list of primers used for validation, the identification, GO and KEGG analysis of DEG and AS events.

**Specifications Table**

**Table:** 

Subject area	Biology
More specific subject area	Cell biology
Type of data	Text file and table
How data was acquired	Affymetrix Human Transcriptome Array 2.0 (HTA 2.0) system
Data format	Analyzed
Experimental factors	Human cartilage endplate-derived stem cells (CESCs) were treated with osteogenic differentiation medium under normoxia and hypoxia respectively.
Experimental features	The total RNA of CESCs in different treatment group was extracted and hybridized to the Affymetrix HTA 2.0. AGCC (Affymetrix GeneChip® Command Console) and EC (Affymetrix Expression Console) software were employed to analyze the data of microarray.
Data source location	Chongqing, China.
Data accessibility	The data is available with this article.

## Value of the data

• The data are valuable for the genome-wide investigation of the mechanisms underlying the differentiation fate of CESCs in response to physiological hypoxia.
• 818 DEGs and 16259 AS events were identified during the CESCs osteogenesis under hypoxia compared with that under normoxia.
• The GO and KEGG (Kyoto Encyclopedia of Genes and Genomes) analysis of DEGs and AS events determined the significantly enriched GO terms (biological process, molecular function and cellular component) and signaling pathways, which is beneficial to the understanding of mechanisms underlying cartilage endplate degeneration.

## 1 Data

The data includes the information of patients from which the clinical samples were obtained, the list of primers, the identification, GO and KEGG analysis of DEG and AS events. The data is available with this article and is related to [1].

## 2 Experimental design, materials and methods

### 2.1 Experimental design

Human cartilage endplate-derived stem cells (CESCs) were isolated and treated with osteogenic differentiation medium under normoxia and hypoxia respectively. The total RNA of CESCs in different treatment group was extracted and hybridized to the Affymetrix HTA 2.0. AGCC (Affymetrix GeneChip® Command Console) and EC (Affymetrix Expression Console) software were employed to analyze the data of microarray.

## 3 Materials and methods

### 3.1 CESCs isolation and culture

The CEP tissues were obtained from 6 patients who underwent discectomy and fusion surgeries in the department of orthopedics of Xinqiao Hospital (Supplementary Table 1). The CEPs were minced into small pieces and then digested with 0.2% collagenase II (Sigma, USA) in DMEM/F12 medium (Hyclone, USA) containing 1% fetal calf serum (FCS) overnight at 37 °C. The suspended cells were then filtered through a 70-μm cell filter to remove large aggregates. The suspension was centrifuged at 1000 rpm/min for 5 min. The cell pellet was resuspended in culture medium containing DMEM/F12, 1% penicillin–streptomycin and 10% FCS. Finally, the CEP cells were transferred to a 25-cm^2^ cell culture flask and cultured at 37 °C and 5% CO_2_ condition. After the first passage of expansion, cells were transferred to agarose selection solution. The agarose selection system was used as previously described [2]. Briefly, a 60 mm-diameter sized culture dishes (Costar Corning, USA) were coated with 1% low melting point agarose. Then, 0.5 ml 2% low melting point agarose, 0.5 ml DMEM/F12 medium and 1 ml culture medium containing 5×10^4^ CEP cells was transferred to the culture dishes and cultured in a humidified atmosphere with 5% CO_2_ at 37 °C. The culture medium was changed twice a week. After 6 weeks, cell aggregates of diameter larger than 50 μm were isolated with a sterile pasteur pipette and then transferred to a 25 cm^2^-culture flask. Passage 3 cells were used in this study.

### 3.2 Induction and oxygen deprivation

For chondrogenic differentiation, cells were induced in chondrogenic induction medium (Cyagen, USA). The induction medium was changed twice a week to a 21-day period. For hypoxic culture, CESCs were cultured in 1% O_2_ condition. For normoxic culture, CESCs were cultured in 21% O_2_ condition.

### 3.3 Affymetrix Human Transcriptome Array 2.0

CESCs were induced into chondrogenic differentiation under normoxia and hypoxia respectively. After 21 days, cells were treated with Trizol. Total RNA was extracted and hybridized to HTA 2.0 from Affymetrix Corporation. With probes targeting exons and junctions within genes, the HTA 2.0 can analyze gene expression and AS information simultaneously. The hybridization, scanning of microarray were performed according to the recommended protocols by CapitalBio Corporation (Beijing, China). Briefly, the fluorescence signals of the microarray was obtained as DAT files. The Affymetrix GeneChip® Command Console (AGCC) software converted DAT image signals into CEL digital signals. Next, Affymetrix Expression Console (EC) software was used to treat the CEL files through Robust Multichip Analysis (RMA) algorithm, including probeset signal integration, background correction and quantile normalization. Then the chp files were sent to Affymetrix Transcriptome Analysis Console software to analyze differentially expressed genes (DEGs) and alternatively spliced genes (ASGs). To identify the significantly enrichment of gene ontology (GO) terms and functional pathways, DAVID (http://david.abcc.ncifcrf.gov/tools.jsp), Kyoto Encyclopedia of Genes and Genomes (KEGG, http://www.genome.jp/kegg/), and Molecule Annotation System (MAS) were used. The identification, GO and KEGG analysis of DEGs were given in the Supplementary Tables 3–5. And the identification, GO and KEGG analysis of AS events were given in the Supplementary Tables 6–10.

### 3.4 Criteria for detecting DEGs and ASGs

The relative fold change of gene expression was normalized using induced samples under normoxia as control values. Gene expression fold change ≤−2 or ≥2 was set as default filter criteria for significant DEGs. Spicing index (SI), representing the ratio of exon signal intensity to target gene signal intensity between two groups, was used to identify the ASGs and analyze the exon inclusion/exclusion level. SI value was calculated in following formulas:

$$Normalized\;Intensity{\left(i,j\right)}_{A}[NI{\left(i,j\right)}_{A}]=\frac{exon_{i}\;signal\;intensity\;\mathrm{in}\;\mathrm{condition}\;A}{gene_{j}\;signal\;intensity\;\mathrm{in}\;\mathrm{condition}\;A}$$

$$SI(X,Y)={Log}_{2}\frac{NI(X,Y)H}{NI(X,Y)N}$$

The NI(*i*,*j*)*A* represented the ratio of *i*-th exon signal intensity to the *j*-th gene signal intensity in condition *A*. The footnote *N* stood for normoxic induction condition; the footnote *H* represented hypoxic induction condition. The default filter criteria was set as SI ≥2 or ≤−2 (linear).

### 3.5 Quantitative polymerase chain reaction (qPCR)

The qPCR analysis was performed to validate the results of DEGs during chondrogenic differentiation under normoxia and hypoxia in CESCs. RNA was obtained via Trizol extraction and then transcribed into cDNA using PrimeScriptTM RT Master Mix Kit (Takara, Japan). The SYBR Premix Ex TaqTM II (Takara, Japan) were used for qPCR. The cycle parameters of the reaction were 95 °C for 30 s, 40 cycles of 95 °C for 5 s and then 60 °C for 34 s. The expressions of each gene were normalized to Actin-β expression. The primers were designed and listed in Supplementary Table 2.

### 3.6 Semi-quantitative RT-PCR

Semi-quantitative RT-PCR was employed to identify the ASGs. Total RNA was extracted and then transcribed into cDNA as previously mentioned qPCR methods. The primers of the candidate genes were designed on the constitutively expressed exons flanking the target exons with the Primer Premier software. The expression of each gene was normalized to Actin-β expression. The genes of interest for ASGs validation were selected according to following criteria: (1) Whole exon gain/skip was firstly considered. (2) Higher absolute value of SI was privileged. (3) First and last alternative splicing exons were not included because of the primer design difficulties. The primers were listed in Supplementary Table 2.

### 3.7 Statistical analysis

The data were expressed as the means±SD. Comparative analysis were made by independent-samples *t* test to determine the significance between groups. Significance values were set at P-value of <0.05.

## Footnotes

Appendix A Supplementary data associated with this article can be found in the online version at doi:10.1016/j.dib.2016.04.037.
